# Functional repair of p53 mutation in colorectal cancer cells using trans-splicing

**DOI:** 10.18632/oncotarget.2988

**Published:** 2014-12-10

**Authors:** Xingxing He, Jiazhi Liao, Fang Liu, Junwei Yan, Jingjun Yan, Haitao Shang, Qian Dou, Ying Chang, Jusheng Lin, Yuhu Song

**Affiliations:** ^1^ Institute of Liver Diseases, Tongji Hospital, Tongji Medical College, Huazhong University of Science and Technology, Wuhan, China; ^2^ Institute of Hematology, Union Hospital, Tongji Medical College, Huazhong University of Science and Technology, Wuhan, China; ^3^ Department of Gastroenterology, Union Hospital, Tongji Medical College, Huazhong University of Science and Technology, Wuhan, China

**Keywords:** colorectal cancer cells, mutant p53, trans-splicing

## Abstract

Mutation in the p53 gene is arguably the most frequent type of gene-specific alterations in human cancers. Current p53-based gene therapy contains the administration of wt-p53 or the suppression of mutant p53 expression in p53-defective cancer cells. We hypothesized that trans-splicing could be exploited as a tool for the correction of mutant p53 transcripts in p53-mutated human colorectal cancer (CRC) cells. In this study, the plasmids encoding p53 pre-trans-splicing molecules (PTM) were transfected into human CRC cells carrying p53 mutation. The plasmids carrying p53-PTM repaired mutant p53 transcripts in p53-mutated CRC cells, which resulted in a reduction in mutant p53 transcripts and an induction of wt-p53 simultaneously. Intratumoral administration of adenovirus vectors carrying p53 trans-splicing cassettes suppressed the growth of tumor xenografts. Repair of mutant p53 transcripts by trans-splicing induced cell-cycle arrest and apoptosis in p53-defective colorectal cancer cells *in vitro* and *in vivo*. In conclusion, the present study demonstrated for the first time that trans-splicing was exploited as a strategy for the repair of mutant p53 transcripts, which revealed that trans-splicing would be developed as a new therapeutic approach for human colorectal cancers carrying p53 mutation.

## INTRODUCTION

TP53 (also known as p53), a tumor suppressor gene, plays a central role in apoptosis, cell cycle arrest, senescence, DNA repair, cell metabolism, and autophagy [1, 2]. Mutation in TP53 is arguably the most frequent type of gene-specific alterations in human cancers. p53 mutations are present in more than 50% of all cancers, which contributes to tumorigenesis [3]. Mutant p53 does not induce the expression of wild-type (wt) p53 downstream genes because of the loss of the ability to bind to p53 response elements [3, 4]. In addition, the mutant proteins acquire a “gain-of-function” defined as the ability to augment cell proliferation in the absence of endogenous wt-p53, which promotes the development of cancer [3, 4]. Taken together, p53-targeted therapies should be performed because p53 plays a pivotal role in pathogenesis of human malignancies. A number of strategies have been pursued, including peptides and small molecules that reconstitute p53 tumor suppressor functions in p53-defective cancer cells [5-10]. While, the toxicity of the chemicals limits their applications in humans. p53-based gene therapy had been under investigation, which provided an excellent candidate of p53-targeted therapies. Available p53-based gene therapy as a therapeutic strategy contains the administration of wt-p53 or the suppression of mutant p53 expression in p53-defective cancer cells. The most widely used strategy of p53-based gene therapy is the introduction of wt-p53 cDNA into cancer cells [11-13]. One obvious limitation of this approach is over-expression of wt-p53 which may have adverse effects on normal cells [14, 15]. An alternative approach tried to inhibit the expression of mutant p53 using antisense oligonucleotides, ribozymes and siRNAs [16-19]. Previous researches demonstrated that absent p53 expression increased the risk of cancers due to the loss of wt-p53 function [15, 20]. Therefore, an ideal strategy that simultaneously restores wt p53 production and reduces deleterious mutant p53 expression should be applied in p53-defective cancer cells.

Trans-splicing is a special form of RNA processing in eukaryotes where exons from two different primary RNA transcripts are joined end to end and ligated. This mechanism has been developed as a strategy to correct mutant target mRNAs by splicing an exogenous mRNA into the normal portion of the mutated mRNA [21-23]. This repair is typically achieved by exon replacement and subsequent removal of the defective portion of the target pre-mRNA so that a functional gene product can be transcribed [21-23]. The utility of spliceosome-mediated RNA trans-splicing as an RNA-based technology has been proven *in vitro* studies and in preclinical disease models, including cystic fibrosis (CF) [24-26], haemophilia A [27], and X-linked immunodeficiency with hyper IgM (HIGM1) [28]. It is well known that p53 is mutated in more than 50% of all human cancers including colorectal cancer (CRC) [29]. In theory, trans-splicing can be exploited as a tool for the correction of mutant p53 transcripts in human colorectal cancer cells carrying p53 mutation, which leads to down-regulation of mutant p53 expression and the induction of wt-p53 production. To test the ability of trans-splicing to repair mutant p53 transcripts, the plasmids encoding a pre-trans-splicing molecule (PTM) targeted to p53 intron 7 were delivered into human CRC cells carrying p53 mutation. And the results showed that mutant p53 transcripts was repaired partially by trans-splicing and subsequently resulted in the activation of p53 down-stream target molecules which were responsible for cell cycle arrest and cell apoptosis. Further study revealed that adenovirus vector carrying p53-PTM blocked the growth of tumor xenografts developed by the inoculation of p53-defective CRC cells.

## RESULTS

### Detection of trans-splicing-generated p53 RNA in transfected colorectal cancer cells

**Figure 1:**
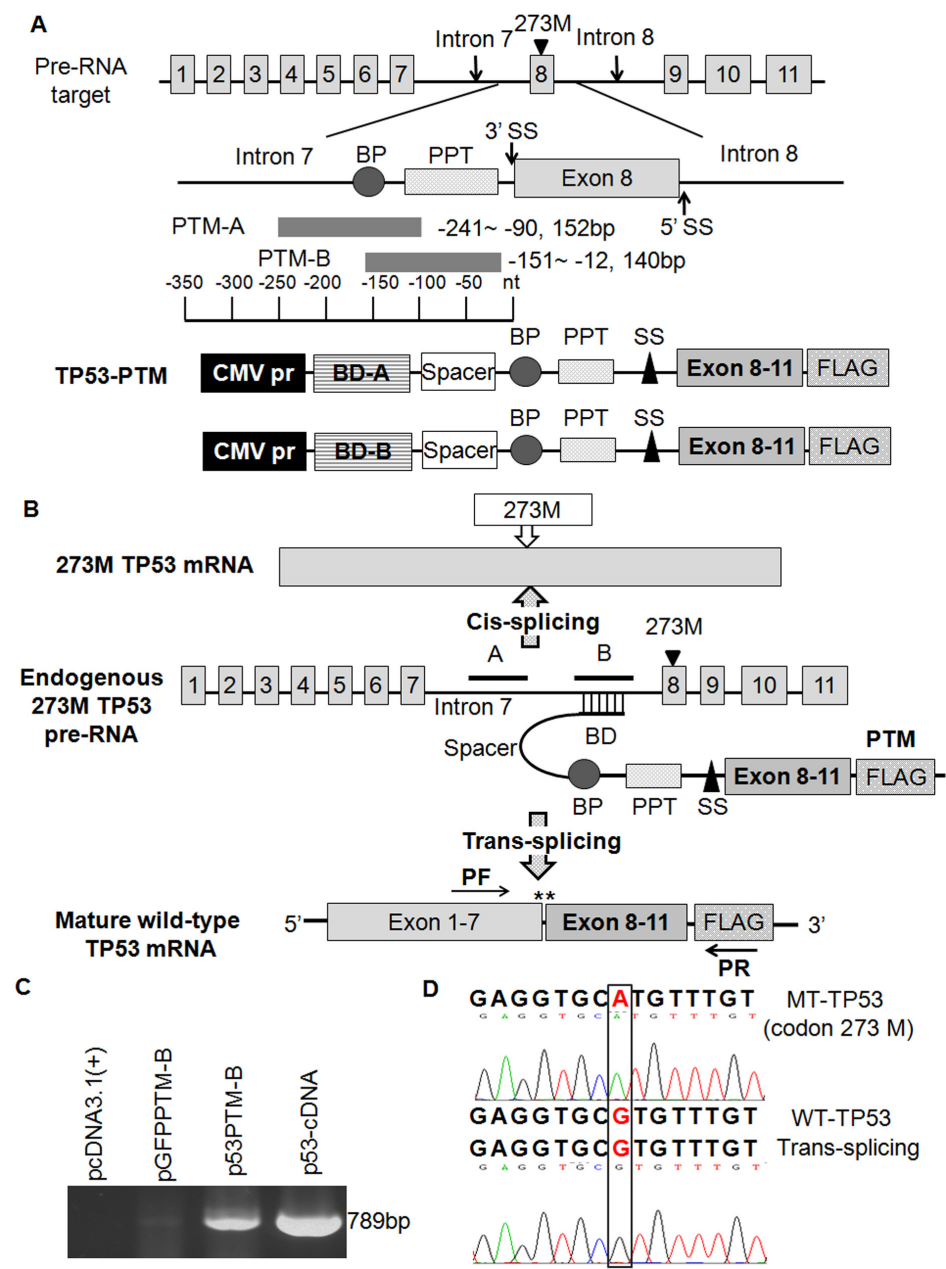
Schematic illustration of trans-splicing used for the correction of mutant p53 transcripts and the detection of trans-spliced p53 RNA in transfected cells A. the structure of p53 pre-trans-splicing molecules (PTM). The hybridization domain is antisense to intron 7 of p53 pre-mRNA. BD-A and BD-B differ only in their binding regions, domain B was more efficient than domain A in promoting *trans*-splicing. BD, binding domain; BP, branch point; PPT, polypyrimidine tract; SS, splicing site. B. Schematic representation of the trans-splicing mechanism. *Cis-*splicing of the mutant p53 pre-mRNA yields a mutant p53 transcript in codon 273. Mutant p53 transcripts were repaired through the approach of trans-splicing. Arrowheads indicate the PCR primers used for detection of trans-splicing-generated products. C. Detection of trans-spliced p53 RNA in transfected HT-29 cells. D. DNA sequence analysis of RT-PCR product of trans-splicing isolated from HT-29 cells transfected with p53-PTM plasmids.

To determine whether trans splicing repaired mutant p53 transcripts in cancer cells, we transfected p53-PTM (Figure 1A) and the controls (Figure S1) into two colorectal cancer cell lines (HT-29 and SW620) carrying p53 mutation in codon 273. Then RT-PCR was performed to detect trans-spliced p53 RNAs using specific primers that bridged the splice junction. To distinguish endogenous p53 transcripts, reverse primer was located in FLAG-tag (Figure 1B). The RT-PCR results demonstrated trans-spliced p53 RNAs were only detected in HT-29 cells transfected with p53-PTM, no detectable products were shown in HT-29 cells transfected with pcDNA3.1 or pGFPPTM (Figure 1C). Not only the amplified product size matched a RT-PCR product generated from intact p53 cDNA (Figure 1C), but also DNA sequence results confirmed trans-splicing-mediated repair of mutant p53 transcripts with high fidelity (Figure 1D). In addition, we demonstrated that mutant p53 transcripts in SW620 cells were also repaired by trans-splicing (Supplementary Figure S2). All these indicated trans-splicing-mediated repair of p53 mutation with high specificity and fidelity. To optimize the efficiency of trans-splicing-mediated correction of mutated p53 transcripts, the trans-splicer constructs with a hybridization domain complementary to different regions of p53 intron 7 were transfected into HT-29 cells, the results of semi-quantitative RT-PCR demonstrated the trans-splicer construct with hybridization domain B (p53-PTM-B) possessed better efficiency (data not shown). Therefore, this construct was used for all subsequent study.

### Induction of cell cycle arrest in human colorectal cancer cells by trans-splicing that repairs mutant p53 transcripts

**Figure 2:**
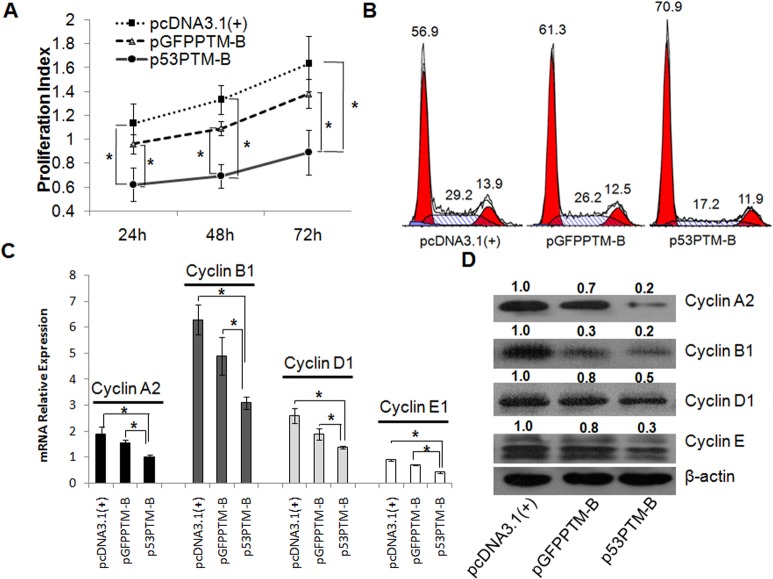
Induction of cell cycle arrest in human colorectal cancer cells (HT-29) through trans splicing-mediated repair of mutant p53 transcripts Trans-splicing plasmids or the controls were transfected into HT-29 cell carrying p53 mutation (codon 273), and then the proliferative activity, cell cycle and cell cycle regulatory genes of HT-29 cells were evaluated. A. MTS assay demonstrated that the proliferative activity of HT-29 cell was inhibited after the introduction of p53-PTM-B. *p<0.05. B. Flow cytometry revealed p53-PTM induces cell-cycle arrest in transfected HT-29 cells. C. Real-time RT-PCR results showed that the regulatory genes for cell cycle in mRNA level decreased significantly in HT29 cells transfected with p53-PTM-B. *p<0.05. D. Expression patterns of the regulatory genes for cell cycle in transfected HT-29 cells were determined by western blot analysis. Densitometric quantification of the indicated proteins was shown. Partial repair of mutant p53 transcripts by trans-splicing resulted in the dramatic decrement in the expression of the regulatory proteins which were responsible for cell cycle.

To determine whether the repaired *p53* transcripts were translated to produce functional p53 protein in transfected cells, we analyzed proliferative activity, the distribution of cell cycle and the expression of regulatory genes responsible for cell cycle in HT-29 cells transfected with p53-PTM or the controls. As shown in Figure 2A, proliferative activity of HT-29 cells was inhibited after the transfection of p53-PTM compared with the controls (p<0.05). Then, we evaluated the effect of p53-PTM on the distribution of cell cycle in HT-29 cell. Cell cycle determined by flow cytometry demonstrated a significant accumulation of the cells in G1 phase following the transfection of p53-PTM into HT-29 cells (Figure 2B). In addition, to further investigate the effect of p53-PTM on cell cycle, we examined the expression patterns of cell-cycle-related genes through quantitative RT-PCR and western blot. As shown in Figure 2C, real-time RT-PCR revealed that partial correction of mutant p53 transcripts by trans-splicing resulted in down-regulation of the mRNA of the genes responsible for the G1/S cell cycle checkpoint such as cyclin A2, B1, D1 and E. In agreement with quantitative RT-PCR data, the similar change in cell-cycle-related proteins was observed in western blot analysis (Figure 2D). All these indicated that p53-PTM induced cell cycle arrest in HT-29 cells through partial correction of mutant p53 transcripts. Simultaneously, the results showed a weak induction of cell cycle arrest in GFP-PTM-transfected CRC cells compared with the negative control (pcDNA3.1) (Figure 2B, C and D).

### Induction of apoptosis in human colorectal cancer cells by trans-splicing that repairs mutant p53 transcripts

**Figure 3:**
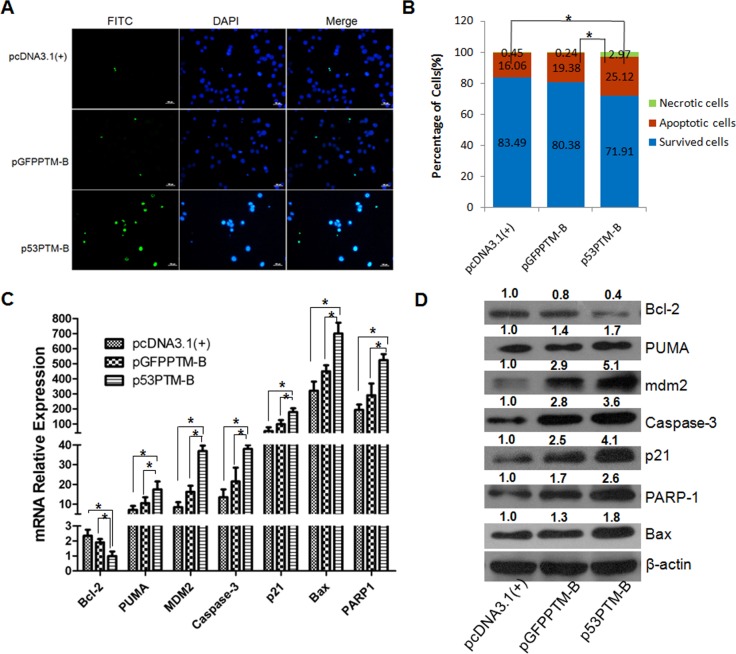
The effect of p53-PTM on the induction of apoptosis in HT-29cells A. TUNEL staining analysis showed p53-PTM induced the apoptosis of HT-29 cells. B. Cell apoptosis of HT-29 cells was analyzed by flow cytometry using Annexin-V/propidium iodide after HT-29 cells were treated by the transduction of p53-PTM. Cell apoptosis was determined by the percentage of apoptotic cell number in total cell number. *p<0.05. C. Real time RT-PCR results showed that the expression of p53-responsive apoptotic genes in mRNA level increased significantly in HT-29 cells transfected with p53-PTM-B. *p<0.05. D. Western blot analysis determined the expression patterns of p53-responsive apoptosis proteins in HT-29 cells upon the transfection of trans-splicing plasmids.

The loss of p53-mediated apoptosis has been implicated as an important event in tumor progression, therefore, we investigated the effect of p53-PTM on the apoptosis. Apoptosis of transfected HT-29 cells was evaluated by TUNEL staining, flow cytometry using Annexin-V/propidium iodide combined labeling and the expression patterns of apoptosis-related genes. As shown in Figure 3A, apoptotic cells detected by TUNEL labeling increased significantly in HT-29 cells following the delivery of p53-PTM (p<0.05). Consistent with the result of TUNEL staining, flow cytometry analysis demonstrated that the percentage of apoptotic cells increased remarkably in HT-29 cells transfected with p53-PTM compared with the controls (Figure 3B). Then, we determined its effects on the expression of apoptosis-related genes in transfected HT-29 cells. Increased expression of the apoptotic pathway genes caspase-3, Bax, PUMA and PARP was detected in HT-29 cell after the delivery of p53-PTM (Figure 3C and D). Simultaneously, the results demonstrated enhanced expression of p53-dependent downstream target gene mdm2 which is responsible for cell survival; up-regulation of p21 involved in growth arrest was confirmed in p53-PTM-transduced HT-29 cells using quantitative RT-PCR analysis and western blot (Figure 3C and D). In addition, the antiapoptotic protein Bcl-2 was reduced in HT-29 cells after mutant p53 transcripts were repaired through trans-splicing. All these revealed that apoptosis was efficiently triggered in HT-29 cells transfected with p53-PTM compared with the negative control (pcDNA3.1).

### p53-PTM reduced the growth of xenograft tumors in nude mice

**Figure 4:**
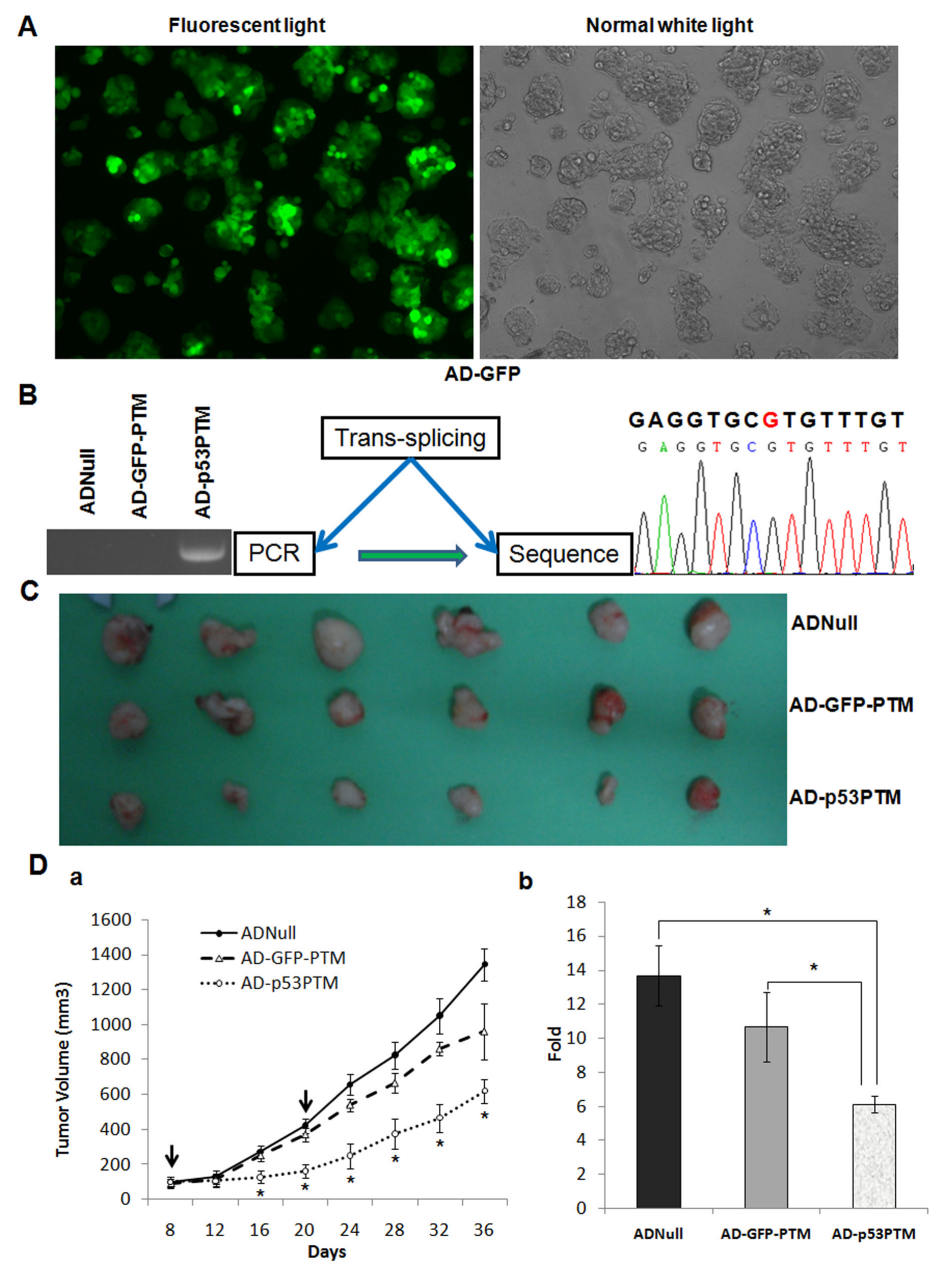
The effect of adenovirus vector expressing p53-PTM on the growth of xenograft tumors in nude mice developed by inoculating HT-29 cells A. Fluorescent microscopy showed efficient transduction of HT-29, as indicated by eGFP expression, 4 days following adenovirus vector administration. B. RT-PCR analysis evaluated trans-splicing-mediated repair of mutant p53 transcripts in xenograft tumors. Adenovirus vectors carrying p53-PTM or the controls were injected into xenograft tumors. 48 hours after injection, RNA were isolated from tumor tissues and then subjected to RT-PCR analysis (left panel) for trans-spliced p53 transcripts. DNA sequence analysis of RT-PCR product (right panel) demonstrated mutant site of p53 in codon 273 was repaired. C. Effects of p53-PTM on the growth of pre-established HT-29 xenografts at a gross morphology level. D. Tumor growth curves (left panel, a) and the average volume fold increase of tumors at the sacrifice with respect to the first measurements (right panel, b) demonstrated antitumor effects of p53-PTM *in vivo*. *p<0.05.

**Figure 5:**
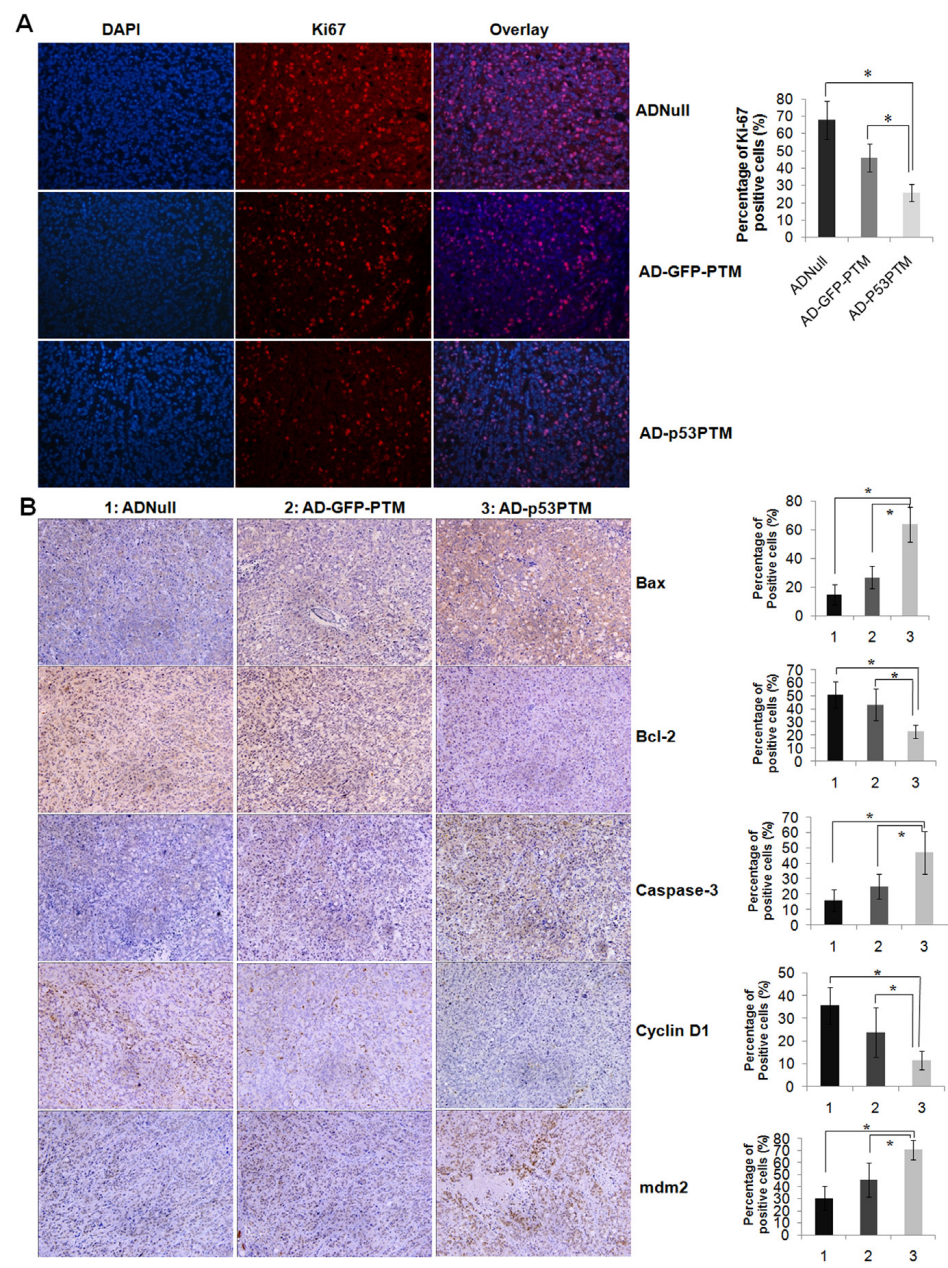
Effect of Ad-p53-PTM on the proliferation, cell cycle and apoptosis of HT-29 cells in xenograft tumors A. Effect of Ad-p53-PTM on the proliferation of HT-29 in pre-established HT-29 xenografts. Cell proliferation was assessed by immnofluorescence staining of Ki67. B. Effects of Ad-p53-PTM on cell cycle and apoptosis in pre-established HT-29 xenografts. Indicated proteins were measured by immunohistochemistry. Representative immunohistochemical staining and the percentage of positive cells in immnostaining were shown. Positive cells were counted in tumor tissues and presented as the mean ± SD (4 random fields per section and three sections per tumor).1: AD-Null, 2: AD-GFP-PTM, 3: AD-p53PTM. * p<0.05.

To determine the effect of p53-PTM on the growth of HT-29 cells *in vivo*, we constructed adenovirus vectors expressing p53 trans-splicing cassettes and delivered them into xenograft tumors developed by the inoculation of HT-29 cells. These adenovirus vectors efficiently transduced HT-29 cells with MOI above 10 (Figure 4A). As a prelude to the analysis of their *in vivo* effects, trans-splicing-mediated repair of mutant p53 transcripts was evaluated in xenograft tumors after intratumoral injection of Ad-p53-PTM or the controls into xenograft tumors. As show in Figure 4B, the results of RT-PCR (left panel) and DNA sequence (right panel) confirmed mutant p53 transcripts was repaired *in vivo* by trans-splicing. To assess the ability of adenovirus-mediated trans-splicing to suppress the growth of tumors *in vivo*, tumor size was monitored over time after the administration of adenovirus vectors. The mice which had received the injection of Ad-p53-PTM showed a marked suppression of xenograft tumors growth, which could be reflected by gross morphology (Figure 4C) and growth curves (Figure 4D). As shown in Figure 5A, the proliferation of CRC cells indicated as ki67 staining was inhibited in tumor tissues of nude mice which received the administration of Ad-p53-PTM. Then, we analyzed the expression of the proteins involved in cell cycle and apoptosis in xenograft tumors to probe the inhibitory mechanism of p53-PTM *in vivo*. Proapoptotic regulatory proteins such as caspase-3 and Bax were barely detectable in tumor tissues of AdNull-treated mice, and augmented remarkably in tumors of mice which received injection of Ad-p53-PTM (Figure 5B). A reduction in cyclin D1, a cell cycle regulatory protein, was observed in tumors of Ad-p53-PTM-treated mice (Figure 5B). In addition, immunohistochemical analysis of tumor tissues showed that the administration of Ad-p53-PTM reduced staining of Bcl-2 identified as antiapoptotic protein (Figure 5B). Enhanced expression of p53-dependent downstream target genes mdm2 was detected in Ad-p53-PTM-treated mice (Figure 5B). These indicated p53-PTM induced p53-depedent downstream target molecules involved in cell cycle arrest and apoptosis, and then reduced the growth of xenograft tumors.

## DISCUSSION

p53 is known to be a tumor suppressor in many tumor types and functions as a powerful transcription factor involved in cell-cycle regulation and apoptosis. Wild-type p53 has a short half-life and its expression level in normal conditions is low. Mutations of p53 gene are arguably the most frequent type of gene-specific alterations in human cancer. Mutant p53 is more stable and can be detected at high levels in tumor cells [3, 30, 31]. Structurally altered mutant p53 transcripts cannot bind to DNA and lose their abilities to trans-activate target genes. More important, mutant p53 isoforms exert dominant–negative effects over co-expressed wt-p53; and mutant p53 proteins acquire “gain-of-function” which contributes to various aspects of tumor progression [3]. In this study, we demonstrated for the first time that the correct 3′ region of p53 mRNA was spliced into the normal 5′ portion of the endogenous RNA using trans-splicing in colorectal cancer cells containing p53 mutation. Then, we provided functional evidences for trans splicing-mediated functional repair of endogenous mutant p53 transcripts, which was revealed by cell-cycle arrest and apoptotic induction in transfected CRC cells. After testing the feasibility of the strategy *in vitro*, the trans-splicing cassettes were packaged into adenovirus, and then adenovirus vectors were injected into tumors created by inoculating HT-29 cells into nude mice. The *in vivo* data showed that adenoviral vectors expressing p53-PTM reduced the growth of xenograft tumors in nude mice through the induction of p53-depedent downstream genes. All these indicated that trans-splicing-mediated repair of mutant p53 transcripts at RNA level resulted in functional correction of p53 mutation *in vitro* and *in vivo,* and thus it provided a useful strategy to engender the production of functional p53 activity in cancer cells to combat cancer.

The widely used strategy of p53-based gene therapy focused on gene replacement based on wt-p53 administration. Although previous studies revealed their effectiveness in cell culture and in animal models [13, 32, 33], aberrant expression of wt-p53 by conventional gene replacement strategies may lead to unintended phenotypic changes in both tumorigenic and normal cells [14, 20, 30]. Increased p53 activity results in cell-cycle arrest, senescence, early differentiation, or apoptotic cell death. Loss of mdm2 or mdm4 in knockout mice leads to embryo lethal phenotypes that are caused by constitutive activation of p53 [20]. In addition, Wang Y *et al* demonstrated that restoring expression of wt-p53 suppressed tumor growth, but did not cause tumor regression in mice with a p53 missense mutation [34]. Alternative strategy is the reversion of mutant p53 phenotype through the inhibition of mutant p53 expression using antisense oligonucleotides, ribozymes and siRNAs. In principle, mutant p53 transcripts were efficiently suppressed at the pre-mRNA or mRNA levels through above techniques. However, absent p53 expression by loss of both p53 alleles or wt-p53 allele gave rise to tumors, or increased the risk of cancer due to loss of wt-p53 function [14, 20]. In this study, an engineered pre-mRNA trans-splicing molecule binds specifically to mutant p53 transcripts in the nucleus; with spliceosome-mediated *trans*-splicing to the pre-mRNA product, the mutant transcript would be partially repaired into the corrected mRNA product. Trans-splicing has several advantages over conventional gene therapy. Trans-splicing can simultaneously reduce mutant p53 transcripts and induce the production of a functional wt-p53 protein, which leads to desired phenotypic changes in p53-defective cancer cells. As the gene is repaired rather than introduced, the spatial and temporal expression of the gene should be controlled by endogenous regulation such that protein expression resembles that of normal individuals. In addition, the repair by spliceosome-mediated *trans*-splicing only occurs where the target transcript is expressed, adverse effects would not be anticipated in normal cells where low level of functional p53 transcripts exist. Another advantage is that the PTM constructs are easily accommodated in current vector systems which have packaging limitation because only a fragment of the gene should be contained in trans-splicer. Thus, trans-splicing represents a potentially approach for the treatment of p53-defective cancers because it offers some unique advantages over currently used techniques in p53 manipulation.

While, there are some limitations of trans-splicing in its application to p53-mutated cancer. Firstly, low efficiency would be a major disadvantage for the application of *trans-*splicing to human diseases. Only part of mutant p53 transcripts were corrected by *trans-*splicing. Fortunately, our results demonstrated CRC cells transfected with p53-PTM exhibited phenotypic alteration. Repair of mutant p53 transcripts through trans-splicing was enough to induce apoptosis and cell cycle arrest in CRC cells, and suppress the growth of xenograft tumors. Secondly, a single pre-trans-splicing molecule do not repair all mutations in defective gene. In this study, p53-PTM-E8-11 only repaired p53 mutation in exon 8-11. So different trans-splicing constructs should be created for cancer cells carrying diverse p53 mutation.

Colorectal cancer (CRC) is the third most commonly diagnosed cancer in males and the second in females, with over 1.2 million new cancer cases and 608,700 deaths estimated to have occurred in 2008 worldwide [35]. The majority of p53 mutations identified in colorectal cancer were missense mutation. Most of p53 mutations were concentrated in the regions encoding p53 DNA binding domain, codon 175, 248 and 273 are considered mutation hotspots [36, 37]. In this study, human CRC cell lines (HT-29 and SW620) carrying p53 mutation in codon 273 are used as experimental cell models for p53-targeted therapy. Compared with negative control (pcDNA3.1), the plasmid encoding p53-PTM inhibited the growth of p53-mutated CRC cells remarkably *in vitro*. The *in vivo* data also showed Ad-p53-PTM suppressed the growth of xenograft tumors significantly. p53 pre-trans-splicing molecules repaired mutant p53 transcripts, which resulted in a reduction of mutant p53 transcripts and an induction of wt-p53 transcripts simultaneously. Our study provided an excellent example for the feasibility of trans-splicing-mediated repair of mutant p53 transcripts in p53-defective cancers; therefore, trans-splicing will be a possible candidate for the use in future effective therapy against p53-defective cancers. In addition, we showed a weak inhibitory effect of GFP-PTM on the growth of HT-29 cells *in vitro* and *in vivo* compared with the negative control (pcDNA3.1 or AdNull). GFP-PTM was delivered into p53-mutated CRC cells, resulting in the replacement of mutant p53 transcript with GFP acceptor through trans-splicing. Therefore, GFP-PTM reduced mutant p53 transcripts without the induction of wt-p53. The effect of GFP-PTM on the phenotype of CRC cells was attributed to a reduction in mutant p53 transcripts.

In conclusion, our findings demonstrated for the first time mutant p53 transcripts was repaired using trans-splicing in CRC, which resulted in phenotypic correction of p53-defective CRC cells *in vitro* and *in vivo*. These reveal that trans-splicing will be provided as a new therapeutic approach for cancers carrying p53 mutation. However, the improvements in the efficacy of trans-splicing and gene transfer systems will likely be required in further study.

## METHODS

### Plasmid construction

The structure of p53 PTMs illustrated in Figure 1 included the cytomegalovirus immediate-early promoter, a hybridization domain (also known as binding domain, BD), spacer, branch point (BP), polypyrimidine tract (PPT) and splice acceptor site followed by Exon 8-11 and Flag-tag. Exons 8-11 of human p53 cDNA were obtained by PCR from the plasmid pCMV-Neo-Bam wt-p53 (kindly provided by Prof. Bert Vogelstein, Johns Hopkins University School of Medicine). Previous research [38] and our preliminary study demonstrated that the optimal size of the hybridization domain for maximal efficiency of trans-splicing was about 150 nt. To determine the optimal hybridization location for maximal efficiency of *trans*-splicing, two hybridization domains derived from *Homo sapiens* p53 intron 7 (GenBank accession NG_017013.2 ) were tested (see Figure 1A, labeled BD-A or B); hybridization domain “A” included nt −241 to −90; domain “B” nt −151 to −12. DNA sequences of spacer, branch point, polypyrimidine tract and splice site were described as previously [25, 27, 28, 39]. The spacer was comprised of a *de novo* sequence (5′-GCGGCCGCC CGAATAA GTGATTGA TTGAG TTT-3′), that is non-homologous to any murine or human sequence. This was followed by the artificial branch point and polypyrimidine tract and splice acceptor site (5′-TACTAACTGAT ATCTCT TCTTTTTTTTTTT CCGGA AAACAG-3′). The structure of pGFP-PTM (Figure S1) resembled that of p53-PTM, the only difference between them was that eGFP acceptor (GenBank accession # U55763.1 nt 995-1410) in pGFP-PTM replaced exons 8-11 of p53 cDNA in p53-PTM. eGFP acceptor in pGFP-PTM was amplified by PCR from the plasmid pEGFP-C1. For the plasmid expressing p53 cDNA, p53 cDNA obtained by PCR from pCMV-Neo-Bam wt-p53 was cloned into pcDNA3.1(+) vector backbone. All plasmids for *in vitro* experiments were based on a backbone derived from the pcDNA3.1(+) vector (Invitrogen, Carlsbad, CA). All the primers used for plasmids construction were listed in supplementary Table S1.

### Cell culture and cell transfection

Human colorectal cancer cell lines HT29 (ATCC HTB-38™) and SW620 (ATCC CCL-227™) were cultured in DMEM, supplemented with 10% fetal bovine serum, 100 units/ml of penicillin, and 100 μg/ml of streptomycin at 37°C in a humidified atmosphere of 5% CO2. There is a G to A mutation in codon 273 of p53 gene in HT29 and SW620 cells. The plasmids were transfected into the cells using lipofectamine^TM^ 2000 (Life Technologies, Gaithersburg, MD, USA). 48 hours after transfection, the transfected cells were collected for further study.

### Evaluation of trans-splicing-generated p53 RNA in transfected cells carrying mutant p53 transcripts

Total RNA was isolated from transfected cells with Trizol (Invitrogen) and reverse transcribed using the First Strand cDNA Synthesis Kit (Fermentas, Burlington, VT, USA). To detect trans-splicing products in transfected cells, the reverse transcribed cDNA was subjected to PCR amplification using forward primer (PF) specific to exon 5 and reverse primer (PR) specific to Flag-tag. The purified PCR products were sequenced to confirm their fidelity in the repair of mutant p53.

### Analysis of the function of trans-spliced p53 *in vitro*

### Quantitative real-time PCR analysis

Total RNA isolated from transfected cells was reverse transcribed into cDNA, and then real-time PCR was performed using the Platinum^®^ SYBR^®^ Green qPCR SuperMix UDG reagent (Invitrogen, Carlsbad, CA, USA) and ABI 7900HT Fast Realtime PCR System (Applied Biosystems). Primers used were listed in supplementary Table S2.

### Western blot

Protein extraction and western blot analysis were performed as previously described [39].

Cells were harvested for protein extraction 48h after transfection. Lysates from transfected cells were collected using RIPA buffer (Sigma R0278, St Louis, MO, USA). Protein lysates were separated on SDS-polyacrylamide gels, electroblotted onto polyvinylidene difluoride membrane and immunoblotted with primary antibodies, then with peroxidase-conjugated secondary antibodies. After three additional washes, the immune complex was detected by ECL detection (Thermo 34077; Rockford, IL, USA). The following antibodies were used: anti-Cyclin A2 (1540-1, Epitomics, Burlingame, CA, USA), anti-Cyclin B1(1495-1, Epitomics), anti-Cyclin D1(1677-1, Epitomics), anti-Cyclin E(4129;Cell Signaling Technology, Beverly, MA, USA), anti-Bcl-2 (1017-1, Epitomics), anti-PUMA(1652-1, Epitomics), anti-mdm2 (556353, BD PharMingen, San Diego, CA, USA), anti-Caspase-3 (1087-1, Epitomics), anti-p21(2990-1, Epitomics), anti-PARP-1 (1072-1, Epitomics), anti-Bax (1063-1, Epitomics), anti-β-actin (60008-1-Ig, Protein Tech, Chicago, IL, USA), goat anti-mouse IgG-HRP (sc-2005, **Santa Cruz Biotechnology,** Dallas, Texas) and goat anti-rabbit IgG-HRP (sc-2004, Santa Cruz **Biotechnology**).

### Cell proliferation assay and flow cytometry analysis of cell cycle and apoptosis

These assays were detected as we have described before [40].

### TUNEL Staining

HT-29 cells grown on chamber slides were transfected with p53-PTM or the controls. After 48 hours, TUNEL Staining was performed by terminal deoxynucleotidyl transferase-mediated nick end labeling staining (In Situ Cell Death Detection kit, Fluorescein, Roche Diagnostics, Mannheim, Germany). Nuclei were stained with 4,6-diamidino-2-phenylindole (DAPI).

### Generation of recombinant adenovirus expressing trans-splicing cassettes and the infection of adenovirus vector

Trans-splicing expression cassettes containing a hybridization domain, spacer, branch point, polypyrimidine tract, splice acceptor site, Exon 8-11 of p53 cDNA and Flag-tag were cloned into shuttle plasmid of AdMax™ system with Cre-lox (Microbix Biosystems Inc.). Recombinant adenoviruses (Ad-p53-PTM) were generated after packed in 293 cells and then purified through Adeno-X™ Virus Purification Kit (BD Biosciences, Clontech). Additionally, Ad-GFP-PTM, an adenovirus only expressing green fluorescent protein (GFP) pre-trans-splicing molecules and AdNull were yielded as the controls. HT-29 cells were infected with adenovirus vectors for 90 min at multiplicity of infection (MOI) of 1, 10, 100, 1000, 10000 respectively.

### Animal experiment

### Tumor xenograft study

BALB/c athymic nude mice (male 5-6 weeks old and 18-20 g) were purchased from Beijing HFK Bioscience Co.LTD (Beijing, China) and bred at pathogen-free conditions. All animal experiments were carried out in accordance with the Guide for the Care and Use of Laboratory Animals of Tongji Medical College. To establish xenograft tumor, 2×10^6^ HT-29 cells suspended in 100 μl DMEM were inoculated subcutaneously into the back of mice. After eight days, the transplanted nude mice were randomly divided into 3 groups (n=6 each). 2×10^8^ TU adenovirus vectors per mouse were administrated with 2 intratumor injections at day 8 and 20. Tumor volume (V) was monitored by measuring the length (L) and width (W) and calculated with the formula V= (L×W^2^) × 0.5.

### Immunohistochemical analysis and immunofluorescence staining

Tumor specimens were fixed in 4% paraformaldehyde, embedded in paraffin, cut into 4 μm pieces and mounted on polylysine-coated slides. Immunohistochemical analysis and immunofluorescence staining were performed as previously described [41, 42].

### Statistical assessment

All data are expressed as mean ± standard error from 3 separate experiments performed in triplicate except otherwise noted. Tests for significance of difference between groups employed the Student t test, where p<0.05 was taken as an indication of a significant difference between groups.

## SUPPLEMENTARY MATERIAL FIGURES AND TABLES


