# Supplementary material for: Functional repair of p53 mutation in colorectal cancer cells using trans-splicing
Source: Oncotarget. 2014 Dec 10;6(4):2034–45. doi: 10.18632/oncotarget.2988 (PMC4385834; doi:10.18632/oncotarget.2988)
Supplement: Supplementary file 1 [file oncotarget-06-2034-s001.pdf]

Functional repair of p53 mutation in colorectal cancer cells using trans-splicing

Supplementary Material

Functional repair of p53 mutation in colorectal cancer cells using trans-splicing

Xingxing He, Jiazhi Liao, Fang Liu, Junwei Yan, Jingjun Yan, Haitao Shang, Qian Dou, Ying Chang, Jusheng Lin, and Yuhu Song

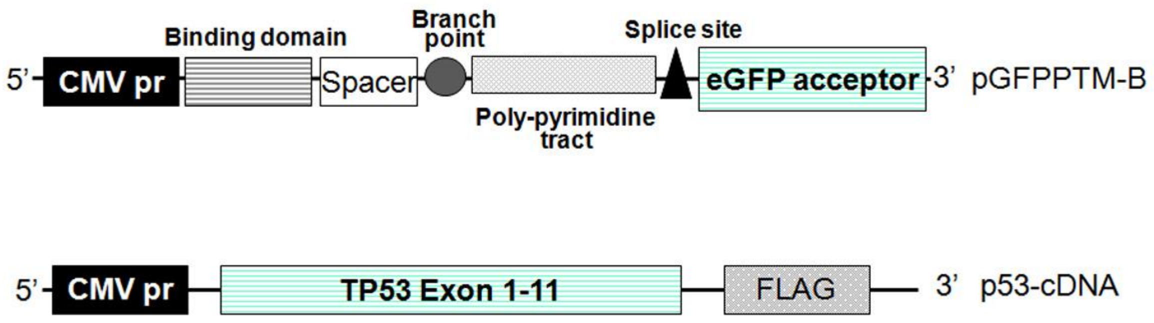

Figure S1: Schematic representation of the plasmids encoding eGFP pre-trans-splicing molecules (PTM) and p53 cDNA. The plasmid (pGFPPTM-B) contained CMV promoter, a binding domain, spacer, branch point, poly-pyrimidine tract, splice acceptor site, eGFP acceptor (GenBank accession # U55763.1 nt 995-1410) and Flag-tag. The binding domain is antisense to intron 7 of p53 pre-mRNA. The plasmid of p53 cDNA contained CMV promoter, p53 cDNA (GenBank accession # NM_000546 nt 198-1379) and Flag-tag, which was used as positive control.

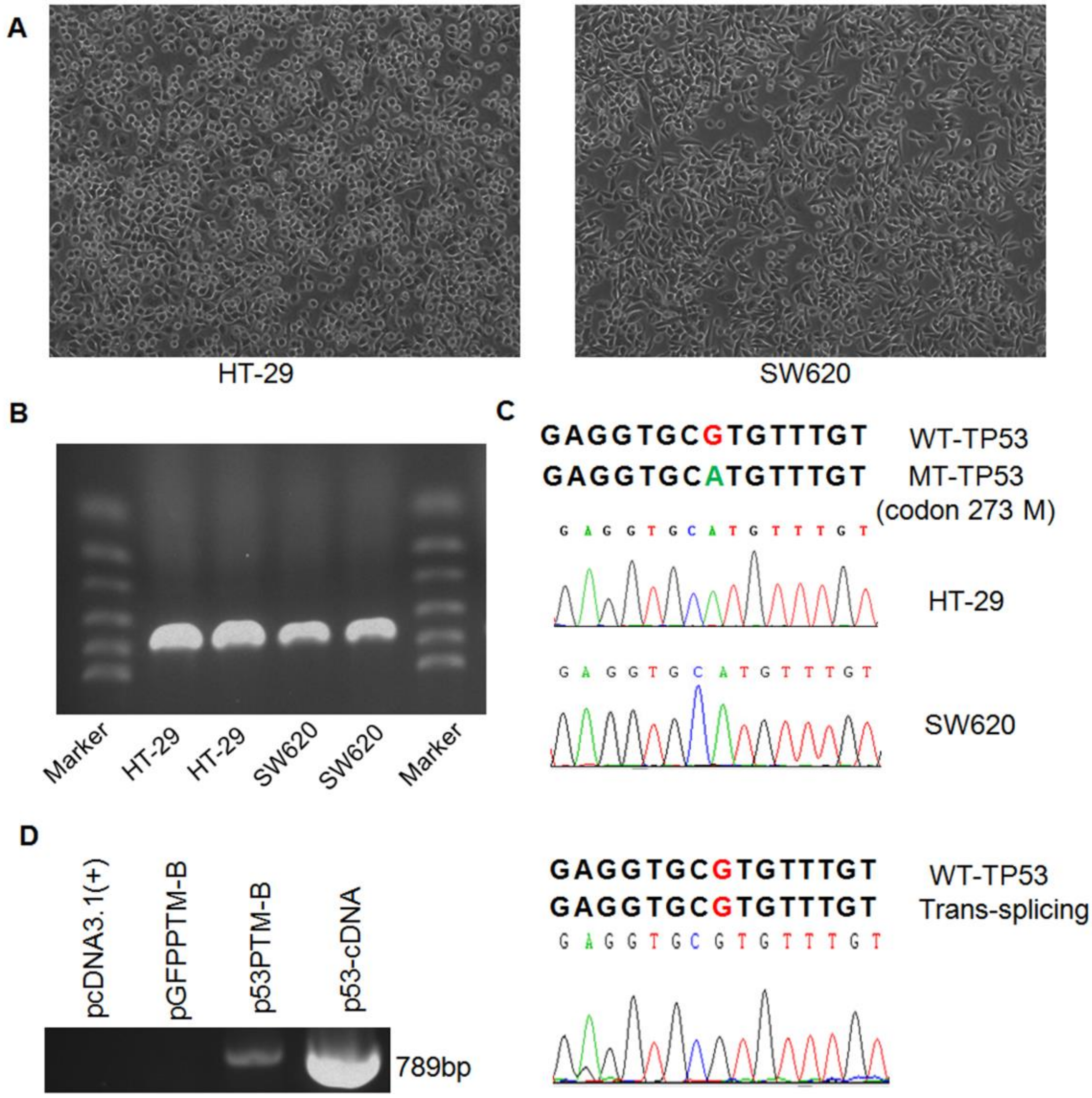

Figure S2: The detection of trans-splicing-mediated repair of mutant p53 transcripts in transfected SW620 cells.

A. The micrographs of colorectal cancer cell lines HT-29 and SW620.

B. RT-PCR (Reverse transcription-polymerase chain reaction) was performed to amplified part of TP53 cDNA on total RNA isolated from colorectal cancer cell lines HT-29 and SW620. The primers used for RT-PCR (TP53-FP and TP53-RP) were listed in Supplementary Table1.

C. DNA sequence results of the RT-PCR products amplified from HT-29 and SW620 cells. The results confirm there is a G to A mutation in codon 273 of the p53 gene resulting in an Arg to His substitution in HT-29 and SW620 cells.

D. Detection of trans-spliced p53 RNA in SW620 cells transfected with p53-PTM-B. The result of RT-PCR showed the generation of trans-spliced p53 RNA in SW620 cells transfected with p53-PTM-B. DNA sequence analysis of trans-spliced p53 RNA confirmed that the mutation in codon 273 of the p53 gene was repaired by trans-splicing.

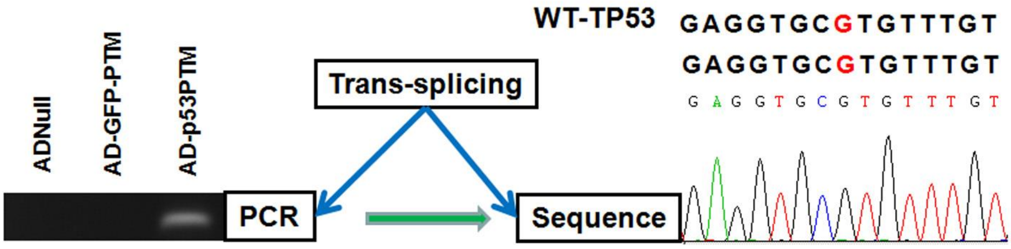

Figure S3: The detection of trans-splicing-mediated repair of mutant p53 transcripts in HT-29 cells infected with adenovirus vector expressing p53-PTM. RT-PCR analysis evaluated trans-spliced p53 transcripts in HT-29 cells infected with Ad-p53-PTM. HT-29 cells were infected with recombinant adenovirus expressing p53-PTM or the controls; 48 hours after infection, RNA isolated from infected cell was subjected to RT-PCR analysis for trans-spliced p53 transcripts. The result demonstrated that trans-spliced p53 mRNA was only detected in HT-29 cells infected with Ad-p53-PTM (left panel). DNA sequence analysis of the RT-PCR product demonstrated mutant site of p53 in codon 273 was repaired (right panel).

Table S1: Primers used for plasmids construct, the detection of trans-splicing and the amplification of p53 cDNA.

Primer names	Purpose	Sequences (5' to 3')
**TP53-FP**	Amplification of p53 part cDNA used for the detection of p53 mutation	CCCCACCATGAGCGCTGCTC
**TP53-RP**	Amplification of p53 part cDNA used for the detection of p53 mutation	CGCTCACGCCCACGGATCTG
**TP53-S-P**	Sequencing primer used for the detection of p53 mutation	ATGAGCCGCCTGAGGTTGG
**BD-A-FP**	Amplification of binding domain A	CGC GGATCC AACCACCCTTGTCCTTTCTG
**BD-A-RP**	Amplification of binding domain A	CCG GAATTC ACCCATCCACCTCTCATCACATCC
**BD-B-FP**	Amplification of binding domain B	CGC GGATCC AGGAAAAGAGAAGCAAGAGGCAGT
**BD-B-RP**	Amplification of binding domain B	CCG GAATTC TGTGGCTTCTCCTCCACCTACCT
**p53-E8-11-FP**	Amplification of exon 8-11 of p53	ATAAGAAT GCGGCCGC TCTTCTTTTTTTTGCAGTGGTAATCTACTGGG
**p53-E8-11-RP**	Amplification of exon 8-11 of p53	CCGCTCGAGCTA**CTTGTCATCGTCGTCCTTGTAGTC**GTCTGAGTCAGGCCCTT
**p53-cDNA-FP**	Amplification of p53 cDNA	CGCGGATCCGCCACCATGGAGGAGCCGCAG
**p53-cDNA-RP**	Amplification of p53 cDNA	CCGCTCGAGCTA**CTTGTCATCGTCGTCCTTGTAGTCGTC**TGAGTCAGGCCCTT
**PF**	Detection of trans-spliced p53 RNA	CAAGACCTGCCCTGTGCAGC
**PR**	Detection of trans-spliced p53 RNA	ACTTGTCATCGTCGTCCTTG

FP, forward primer; RP, reverse primer. BamHI:GGATCC; EcoRI:GAATTC; NotI:GCGGCCGC; XhoI:CTCGAG;

FLAG: **CTT GTC ATC GTC GTC CTT GTA GTC.**

Table S2: Primers used in SYBR Green qRT-PCR.

Primer names	Sequences (5’ to 3’)
**Cyclin A2-FP**	CAGACGGCGCTCCAAGAGGA
**Cyclin A2-RP**	AGGGGTGCAACCCGTCTCG
**Cyclin B1-FP**	TCCGAGTCACCAGGAACTCGAAAA
**Cyclin B1-RP**	AGTCCGGGCTTGGAGGTTGC
**Cyclin D1-FP**	GCGTACCCCGATGCCAACCT
**Cyclin D1-RP**	CCTCGCAGACCTCCAGCATCC
**Cyclin E1-FP**	CAGGGGCGTCGCTGATGAAGA
**Cyclin E1-RP**	TGCTCGGGCTTTGTCCAGCAA
**Bcl-2-FP**	CGGAGGCTGGGATGCCTTTGT
**Bcl-2-RP**	AGCTCCCACCAGGGCCAAAC
**p21-FP**	CACTCAGAGGAGGCGCCATGT
**p21-RP**	GCCCGCCATTAGCGCATCAC
**Bax-FP**	GCAAACTGGTGCTCAAGGCCC
**Bax-RP**	TCTCACCCAACCACCCTGGTCT
**PUMA-FP**	TGGGACTCCTGCCCTTAC
**PUMA-RP**	GGCTGGGAGTCCAGTATG
**MDM2-FP**	GCGCCCCGTGAAGGAAACTG
**MDM2-RP**	TGCACATTTGCCTGCTCCTCAC
**Caspase-3-FP**	GGCGGTTGTAGAAGAGTTTCGTGA
**Caspase-3-RP**	ACCGAAAACCAGAGCGCCGA
**PARP1-FP**	GATTGCTGTGGCACGGGTCC
**PARP1-RP**	TAGCCTGTCACGGGCGCTTC
**GAPDH-FP**	CCCCAGCAAGAGCACAAGAG
**GAPDH-RP**	GCACAGGGTACTTTATTGATGGTAC

FP, forward primer; RP, reverse primer
